# Synthesis of embedded Au nanostructures by ion irradiation: influence of ion induced viscous flow and sputtering

**DOI:** 10.3762/bjnano.5.10

**Published:** 2014-01-29

**Authors:** Udai B Singh, D C Agarwal, S A Khan, S Mohapatra, H Amekura, D P Datta, Ajay Kumar, R K Choudhury, T K Chan, Thomas Osipowicz, D K Avasthi

**Affiliations:** 1 Inter-University Accelerator Centre, Aruna Asaf Ali Marg, New Delhi 110067, India; 2 University School of Basic and Applied Sciences, Guru Gobind Singh Indraprastha University, New Delhi 110078, India; 3 National Institute for Materials Science, Tsukuba, Ibaraki 305-0003, Japan; 4 Institute of Physics, Sachivalaya Marg, Bhubaneswar 751005, India; 5 Nuclear Physics Division, Bhabha Atomic Research Centre, Mumbai, India; 6 National University of Singapore, Singapore

**Keywords:** embedded nanoparticles, ion beam irradiation, recoil implantation

## Abstract

The ion-irradiation induced synthesis of embedded Au nanoparticles (NPs) into glass from islands of Au on a glass substrate is studied in the context of recoiling atoms, sputtering and viscous flow. Cross sectional transmission electron microscopy studies revealed the formation of Au NPs embedded in the glass substrates by the 50 keV Si^−^ ion irradiation of irregularly shaped Au nanostructures on the glass surfaces at a fluence of 3 × 10^16^ ions/cm^2^. The depth profiles of Au in the samples were obtained from high-resolution Rutherford backscattering spectrometry studies. The results from TRIDYN simulation reveal the role of various ion-induced processes during the synthesis of the embedded Au NPs, viz. sputtering and recoiling atoms. Simulation and experimental results suggest that the viscous flow is one of the major factors that are responsible for the embedding of Au nanoparticles into the glass substrate.

## Introduction

Noble-metal nanoparticles (NPs) are of great interest due to their large surface-to-volume ratio and their enhanced absorption of visible light. The shape- and size-dependent properties of nanostructures are used in diverse applications like optical, electronic devices, medical and biological applications [1–5]. It is important to develop reliable fabrication techniques to produce nanostructures with the desired properties and to understand the underlying mechanisms. Several methods are used for the fabrication of nanostructures. Among them, ion irradiation and ion implantation are two well established tools for the synthesis of nanostructures on the surface or embedded NPs [6–11]. The atom beam sputtering technique has been used in our group for the synthesis of embedded Au NPs in a silica matrix [12–16]. Ion implantation is one of the standard ways for the synthesis of noble metal NPs embedded in a matrix and offers the control over the depth distribution of the NPs by properly adjusting the parameters of the ion beam such as ion energy, ion dose, dose rate, and substrate temperature. One of the biggest drawbacks for this procedure is the requirement of a specific ion source to generate ion beams of the desired element. Recoil implantation overcomes this difficulty and is an alternative way to introduce foreign atoms into a solid by means of atomic recoils through a thin surface layer of the desired metal when using an ion beam from only one ion source. When an energetic ion strikes a thin film deposited on a substrate, it loses its energy through a sequence of collisions with the atoms in the film. The recoiling atoms can experience secondary collisions in the thin film, thus generating another series of recoiling atoms and continuing in the same way. In this manner, collision cascades are created and some of the recoiling atoms attain enough energy to move across the film–substrate interface. These recoiled atoms stop at different depths in the substrate depending on their kinetic energy. Simultaneously, during the ion irradiation of the thin film some of the recoiled atoms gain more energy than the surface binding energy of atoms in that film and get sputtered from the surface. Perkins and Stroud [17] have shown that recoil implantation is a useful process to introduce impurities from a thin film of metal into a thin near-surface layer of the substrate. Recoil implantation has also been studied in detail in thin films [18–19]. The ion beam induced sputtering during the irradiation of thin films leads to the formation of surface nanostructures, whereas recoil implantation results in formation of NPs embedded in the substrate. Recoil implantation can be used to tailor desired nanostructures by optimizing the ion beam parameters (energy, fluence and angle of incidence) and thickness of thin films. It is always helpful to know the depth distribution of the recoiling atoms as a function of the layer thickness as well as the ion beam parameters in all these cases. The TRIM (Transport of ions in matter) simulation code including dynamic composition changes (TRIDYN) [20–21] can be used to solve this problem. Stepanov et al. [22] have shown that the implantation of 60 keV Ag ions with fluences of (2–4) × 10^16^ ions/cm^2^ leads to the formation of silver NPs in glass near room temperature. It has been shown that Au atoms get buried [10] during the ion beam irradiation of thin films. Ion beam irradiation that was performed by Hu et al. on Pt NPs on a SiO_2_ substrate indicated a sinking-in of the NPs because of an ion-induced viscous flow [23]. Klimmer et al. [24] have also shown that embedded Au NPs can be synthesized by ion beam irradiation of Au NPs on the surface due to sputtering and ion-induced thermodynamic driving forces for the embedding of the NPs. However the lack of comprehensive studies in this direction limits the understanding of the underlying mechanisms. It requires detailed investigation to understand the role of the different mechanisms in the synthesis of embedded nanostructures by ion irradiation of discontinuous thin films on substrate.

In the present contribution, we report the synthesis of Au nanostructures embedded in glass by a simple approach that employs the ion irradiation of Au thin films on a glass substrate*.* The irradiation results in the formation of nanostructures that are embedded near the surface. These embedded Au nanostructures have great potential for the application as substrates for surface enhanced Raman spectroscopy (SERS). Such a SERS substrate is expected to be reusable due to the embedded nanostructures. TRIDYN [20–21], a binary-collision Monte Carlo simulation in dynamic mode, is used for a better understanding of the recoil implantation while taking into account the required dose and ion energy for a thin film target. The observed formation of embedded Au nanostructures in glass can be recognized as the effects of sputtering, of recoil implantation of Au atoms and of thermodynamic driving forces for embedding of Au NPs, which can result from different surface energies of NPs and the substrate.

## Experimental

The glass substrates used for deposition were thoroughly cleaned with boiling trichloroethylene (TCE), then in boiling acetone, followed by boiling in methanol and a repeated rinsing with deionised water. The substrates were then blown dry with N_2_ gas. Thin films of Au with a thickness of 5 nm were deposited on these substrates by thermal evaporation with a deposition rate of 0.1 nm/s under high vacuum conditions. The vacuum of chamber before and during deposition was 2 × 10^−7^ and 3 × 10^−6^ mbar, respectively. Irradiation was performed by using 50 keV Si^−^ ions to a fluence of 3 × 10^16^ ions/cm^2^ at the low-energy accelerator facility (LEAF) of BARC, Mumbai. In order to determine the metal contents of pristine and irradiated samples, high-resolution Rutherford backscattering spectrometry (HRBS) with 500 keV He^+^ ions at an incident angle of 60° and a scattering angle of 65° and an energy resolution of the detector of about 1 keV was carried out at NUS, Singapore [25]. Cross sectional transmission electron microscopy (XTEM) measurements were performed on pristine and irradiated samples by using a 200 kV field emission TEM (JEM 2100F from JEOL) at TEM station, NIMS, Japan. XTEM samples are prepared by the focused ion beam (FIB) method by using a 30 keV Ga beam in a JEOL JEM 9320 FIB.

## Results and Discussions

**Figure 1:**
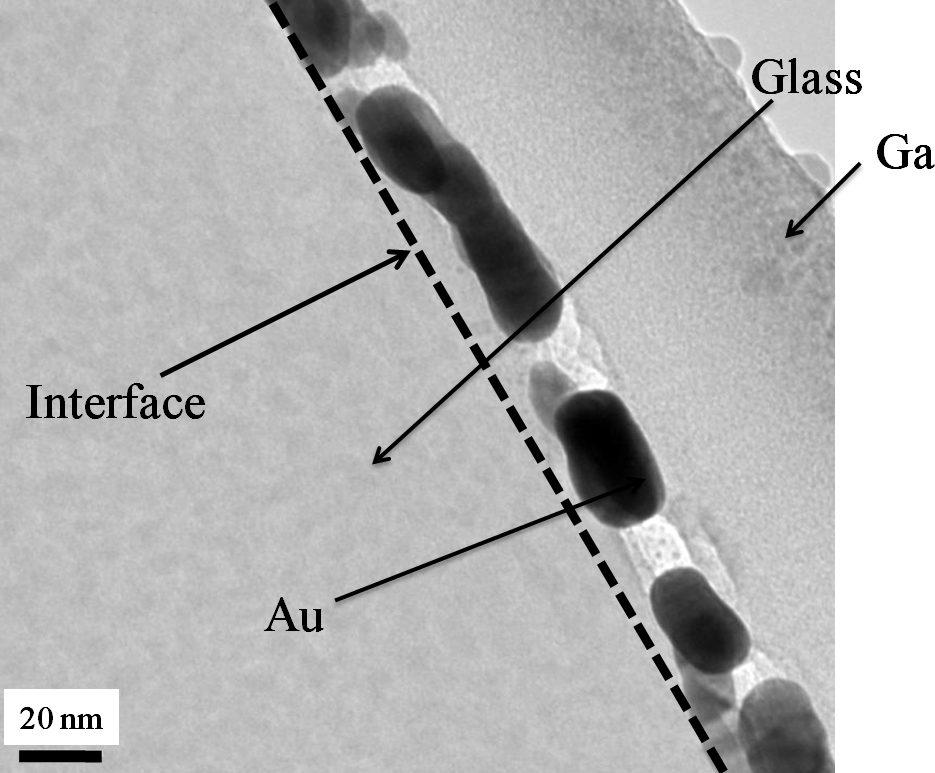
Cross-sectional TEM image of the pristine sample.

**Figure 2:**
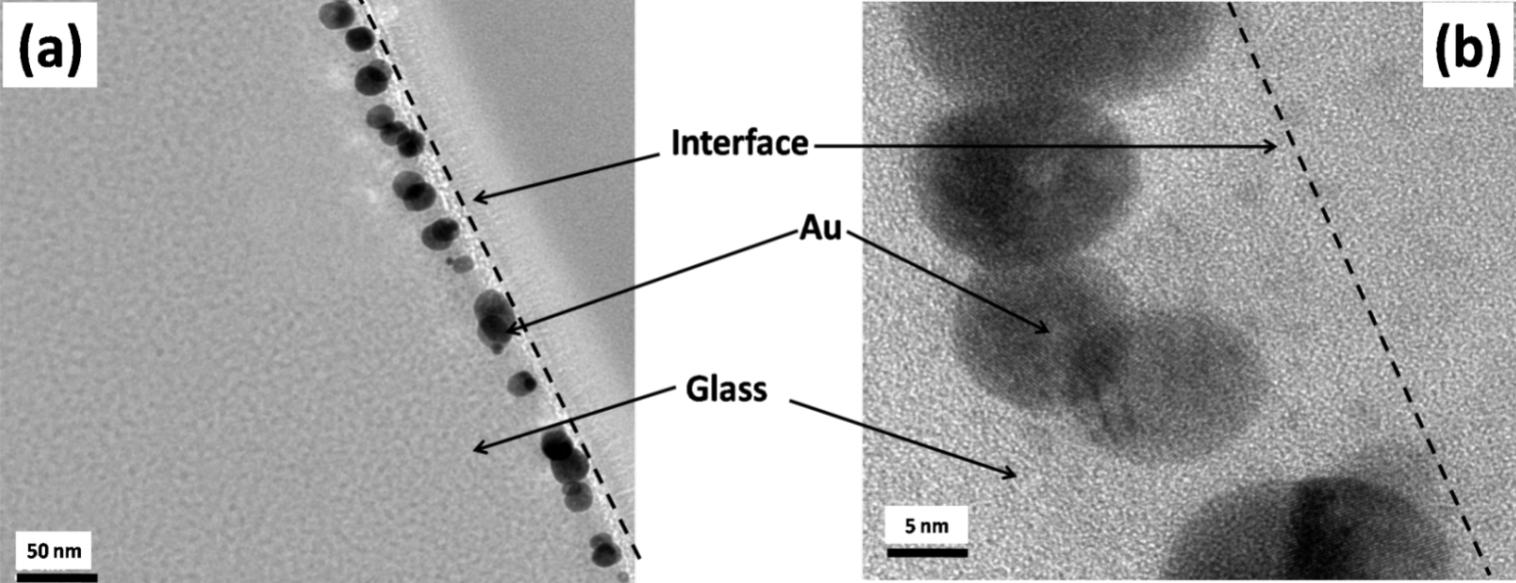
(a) Cross-sectional TEM image of sample irradiated with a fluence of 3 × 10^16^ ions/cm^2^. (b) Higher magnification XTEM image of the near-surface layer.

**Figure 3:**
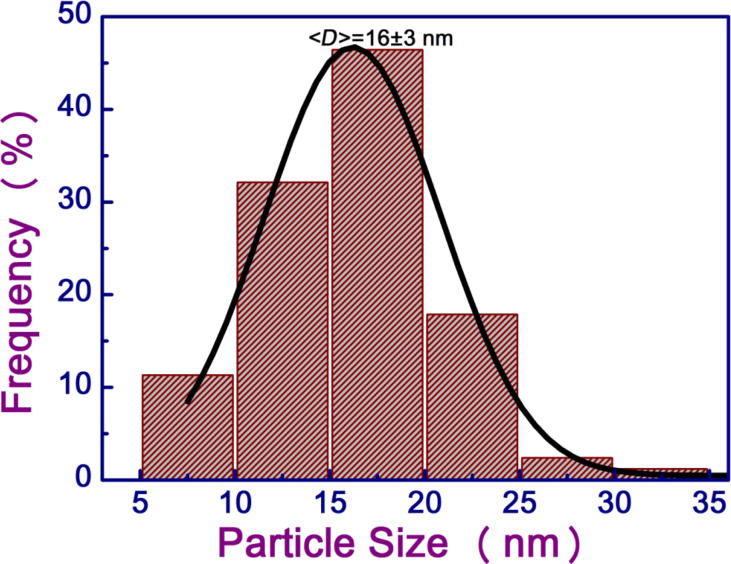
The size distribution of the recoil-implanted Au NPs after the fluence of 3 × 10^16^ ions/cm^2^.

The XTEM image of pristine sample is shown in Figure 1. The image shows that the pristine film was discontinuous with irregularly shaped nanostructures. The glass and Au interface is shown by the dashed line. In this image traces of a few Ga NPs are observed, which originate from Ga that was incorporated during the preparation of the XTEM sample by using focused ion beam. Since the cohesive energy of the metal (Au) is higher than the cohesive energy of the substrate (glass) plus the adhesive energy of the metal on glass, a discontinuous film of Au is formed because of dewetting. The XTEM image (Figure 2) of the sample irradiated with a fluence of 3 × 10^16^ ions/cm^2^ shows the presence of NPs embedded in glass. The size distribution of the embedded NPs is shown in Figure 3. The size of the embedded Au NPs has been found to vary from 5 to 35 nm with an average size of about 16 ± 3 nm.

**Figure 4:**
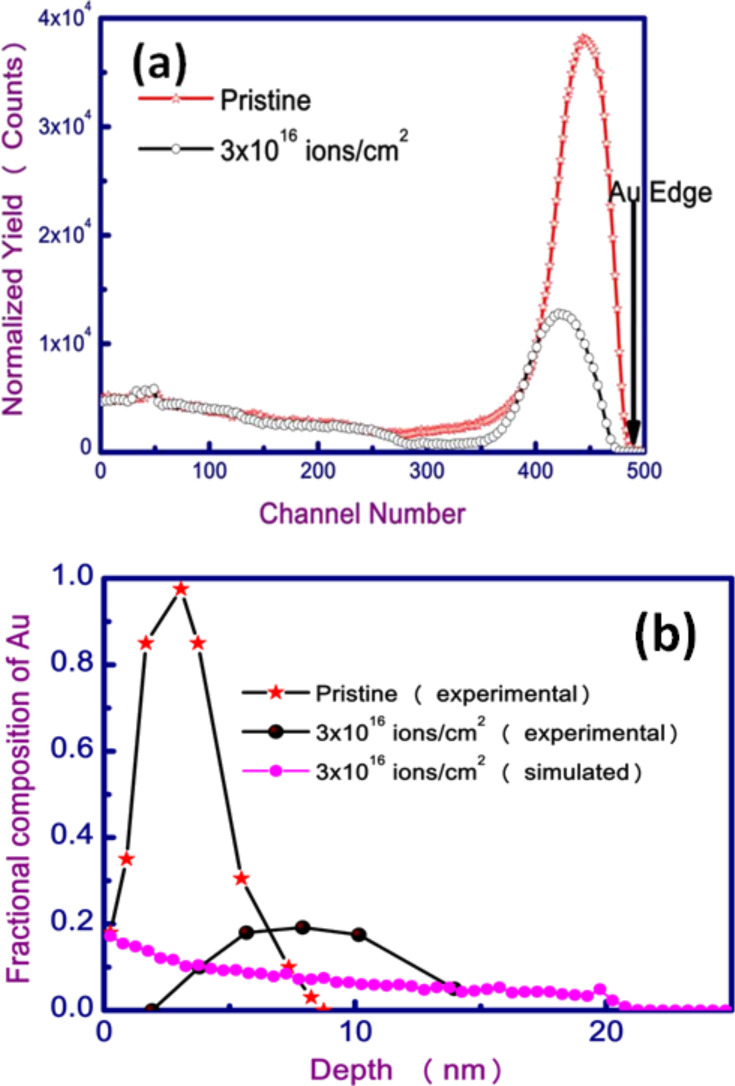
(a) HRBS spectra of pristine sample and sample irradiated with a fluence of 3 × 10^16^ ions/cm^2^. (b) Depth profile of Au in the pristine sample and the sample irradiated with a fluence of 3 × 10^16^ ions/cm^2^ calculated from the HRBS spectra, and Au profile of the irradiated sample simulated with TRIDYN (50 keV Si ion irradiation of Au thin film on a glass substrate).

The HRBS spectra of a pristine sample and a sample irradiated with 50 keV Si ions with a fluence of 3 × 10^16^ ions/cm^2^ are shown in Figure 4a. The reduction in the area of Au peak reveals that the irradiation leads to a decrease in the Au content in the sample due to sputtering. The sputtering yield of Au is estimated to be around 0.4 atoms per incident atom, which is in agreement with the value predicted by the Sigmund theory [26]. The peak of Au in the HRBS spectrum is found to shift to lower energies in the case of the irradiated sample as compared to the pristine sample. This clearly indicates that Au atoms from the thin film get buried into the glass substrate. The depth profiles of Au in both the pristine, the irradiated samples from experimental results and from simulations (which are discussed later) are shown in Figure 4b. The depth profile of Au in the pristine sample shows that the film is discontinuous since the fraction of Au in the film is less than unity. It is observed from the depth profile of the irradiated sample that Au atoms are completely buried into the substrate down to 14 nm from the top layer of the glass substrate.

In the present case, Au NPs embedded in glass are formed due to the ion bombardment on the thin film. The ion irradiation leads to a transfer of momentum from ions to target atoms and during this process the ejection of atoms and the recoil implantation of Au atoms take place. In the recoil implantation process, Au atoms from thin film get buried into the substrate by means of atomic recoil from Au thin film. The efficiency of the recoil implantation depends on the film thickness and ion beam parameters. The number of recoiled metal atoms strongly depends on the thickness of the metal film, and reaches a maximum if the film thickness (*t*) is somewhat smaller than the projected range (*R*_p_ ≈ 20 nm) of the incident ions in the metal film (*t* < *R*_p_ in the present study, from SRIM calculation [27]). The effect of sputtering and recoil implantation on the depth profile of Au in the sample can be simulated by using TRIDYN [27]. TRIDYN takes into account compositional changes of the target due to previously bombarded ions and also modifications of the target. In the TRIDYN simulation, the 50 keV Si ion irradiation with a fluence of 3 × 10^16^ ions/cm^2^ of a Au film deposited on the SiO_2_ substrate (33% Si and 67% O) was performed. Depth profiles of Au in the pristine and the irradiated films (simulated) were obtained as shown in Figure 4b. The total number of recoiled Au atoms from a depth of 8.75 nm to 21.75 nm was 3.44 × 10^15^ atoms/cm^2^ as calculated by the TRIDYN simulation. The existence of Au on the substrate is also observed and the concentration is 5.23 × 10^15^ atoms/cm^2^. The amount of sputtered metal in the simulation is 15.21 × 10^15^ atoms/cm^2^. From the simulation results, it can be clearly observed that the recoil implantation and sputtering take place simultaneously during ion bombardment of Au film. However, it is not in complete agreement with the obtained depth profile of Au from experimental HRBS results, which indicates that some mechanisms are not included in the simulation. NPs on the surface are synthesized because of sputtering and mass redistribution of the thin film that and further irradiation leads to a burrowing of the NPs because of thermodynamic driving forces for the embedding of NPs [24]. These thermodynamic driving forces for the embedding of the nanoparticle may be the processes that are missing from the simulation and which would need to be considered to yield a better agreement with the experimental results.

The synthesis of partially embedded NPs by ion beam irradiation of Pt NPs on SiO_2_ substrate was reported by Hu et al. [23]. This process was explained on the basis of an ion beam induced viscous flow that is created by localized spikes during irradiation. The “local spike” models has been introduced by Bolse [28]. In this model, he has taken into account that the collision cascades of ions of some hundreds of kiloelectronvolts break up into spatially separated subcascades. He introduced a minimum recoil energy, *E*_C_, at which the recoil cascade approximately shows a spherical shape, and the displacement threshold energy, *E*_D_. Elastic thermal spikes should appear when the recoil energy (*E*_R_) falls below the threshold energy (*E*_C_), and local spikes are expected to be initiated at the end of such subcascades, far off the ion track and these recoils whenever *E*_C_ > *E*_D_. In his approach, Bolse [28] calculated the overlap possibility of spherical local spikes and concluded that cylindrically shaped local spikes will form along the subcascade paths by overlapping spherical spikes. Prakash et al. [29] have also reported the formation of Au NPs by Ar-ion induced thermal spikes due to collision cascades that produced molten zones, which led to a dewetting of the Au films on PET through sputtering and crater formation. Thus, ion irradiation of NPs synthesized on the surface leads to a burrowing of the NPs into the substrate [23]. However, in all the mentioned works, not all of the phenomena (sputtering, recoiling atoms and viscous flow) responsible for synthesis of NPs embedded in matrix were considered.

Ion irradiation of Au thin films on glass leads to the formation of Au nanostructures on the surface due to sputtering, dewetting and surface diffusion. It is also observed from TRIDYN simulations that recoil implantation takes place along with sputtering during the irradiation. The total recoil concentration of Au is nearly of the same concentration which is required for the synthesis of embedded NPs through ion implantation reported by Stepanov et al. [22]. Residual metal on the surface leads to the synthesis of nanostructures on the surface. As we observed in our previous report [10], in which the well-ordered nanodots with an average size of 30 nm were created on the surface and were partially buried in the glass substrate after irradiation with a fluence of 1 × 10^16^ ions/cm^2^. In the present study, the average size of nanoparticle decreases to ca. 16 nm with a further increase in fluence as can be seen in Figure 2. Klimmer et al. [24] have reported that the size of the NPs on the surface is decreased with an increase in ion fluence. However, in the present study these NPs are embedded in glass. As we mentioned earlier that thermodynamically driven forces need to be considered in addition to sputtering and recoil implantation in order to quantitatively describe the embedding of Au nanostructures. Ion irradiation results in an effective ion-induced viscous flow of the substrate due to localized elastic thermal spikes [28]. When the ions are hitting surface atoms of a nanoparticle, this primary collision generates a sudden large amount of moving target atoms by secondary knock-on events. The resulting liquid like non-equilibrium state within the nanoparticle depends on the ion beam parameters. Thermodynamic driving forces for embedding of NPs result from the different surface energies, i.e., the surface energy of the particle and its substrate, and the particle–substrate interface energy. It is reported that surface energy of embedded NPs is less than the surface energy of both glass and NPs [23]. The ion bombardment provides the critical kinetic conditions, which are responsible for the embedding of NPs. In the present studiy, the condition *E*_C_ > *E*_R_ > *E*_D_ for generating local spikes due to elastic collision cascades according to the model proposed by Bolse [28] is satisfied since *E*_C_ = 58 eV, *E*_R_ = 45 eV and *E*_D_ = 28 eV in the present case. In this way local spikes are expected to be created by ion irradiation, which may create viscous flow in the glass substrate. Thermodynamic driving forces originated from the surface and interface energies of nanoparticle and substrate are necessary to arrive at an observable ion-induced burrowing effect during ion beam irradiation because the Au nanoparticles want to minimize their surface energy. In this way embedded Au NPs can be created by ion irradiation of Au thin films, which may be an alternative way of ion beam assisted synthesis of embedded NPs after optimizing the thickness of the film and the fluence.

Considering the discrepancy between simulated and experimental depth profiles it appears that the effects of sputtering and viscous flow play a dominant role in the synthesis of embedded Au NPs but the role of recoil implantation may not be completely ruled out. A detailed study should be performed in order to understand the role of recoiling atoms in the synthesis of embedded NPs. In this method, by using one ion source, we can synthesize embedded NPs of any material by optimizing the parameters such as thickness of film, mass and energy of ion.

## Conclusion

Embedded Au nanoparticles (NPs) in a glass substrate are obtained by 50 keV Si ion bombardment on a dewetted thin film of Au deposited on top of that substrate. XTEM and HRBS revealed the formation of Au NPs embedded in glass substrate upon ion irradiation. The synthesis of embedded Au nanostructures is explained on the basis of sputtering, of recoil-implantation and of a viscous flow that is induced by the ion beam. We demonstrated that ion beam induced irradiation of nano-islands on the surface can be used as a technique for synthesis of embedded nanostructures near the surface.

## References

[1] Elghanian R, Storhoff J J, Mucic R C, Letsinger R L, Mirkin C A (1997). Science.

[2] Maier S A, Kik P G, Atwater H A, Meltzer S, Harel E, Koel B E, Requicha A A G (2003). Nat Mater.

[3] Mock J J, Smith D R, Schultz S (2003). Nano Lett.

[4] Ozbay E (2006). Science.

[5] Vasseur J O, Akjouj A, Dobrzynski L, Djafari-Rouhani B, El Boudouti E H (2004). Surf Sci Rep.

[6] Khan S A, Avasthi D K, Agarwal D C, Singh U B, Kabiraj D (2011). Nanotechnology.

[7] Kumar T, Khan S A, Singh U B, Verma S, Kanjilal D (2012). Appl Surf Sci.

[8] Singh U B, Agarwal D C, Khan S A, Kumar M, Tripathi A, Singhal R, Panigrahi B K, Avasthi D K (2011). Appl Surf Sci.

[9] Singh U B, Agarwal D C, Khan S A, Mohapatra S, Tripathi A, Avasthi D K (2012). J Phys D: Appl Phys.

[10] Singh U B, Agarwal D C, Khan S A, Tripathi A, Kumar A, Choudhury R K, Panigrahi B K, Avasthi D K (2011). Radiat Eff Defects Solids.

[11] Khan S A, Srivastava S K, Avasthi D K (2012). J Phys D: Appl Phys.

[12] Avasthi D K, Mishra Y K, Singh F, Stoquert J P (2010). Nucl Instrum Methods Phys Res, Sect B.

[13] Khan S A, Heinig K-H, Avasthi D K (2011). J Appl Phys.

[14] Mishra Y K, Avasthi D K, Kulriya P K, Singh F, Kabiraj D, Tripathi A, Pivin J C, Bayer I S, Biswas A (2007). Appl Phys Lett.

[15] Mishra Y K, Singh F, Avasthi D K, Pivin J C, Malinovska D, Pippel E (2007). Appl Phys Lett.

[16] Mishra Y K, Kabiraj D, Avasthi D K, Pivin J C (2007). Radiat Eff Defects Solids.

[17] Perkins J G, Stroud P T (1972). Nucl Instrum Methods.

[18] Grötzschel R, Klabes R, Kreissig U, Schmidt A (1978). Radiat Eff Defects Solids.

[19] Bruel M, Floccari M, Gailliard J P (1981). Nucl Instrum Methods.

[20] Möller W, Eckstein W, Biersack J P (1988). Comput Phys Commun.

[21] Möller W, Eckstein W (1984). Nucl Instrum Methods Phys Res, Sect B.

[22] Stepanov A L, Hole D E, Townsend P D (1999). J Non-Cryst Solids.

[23] Hu X, Cahill D G, Averback R S (2002). J Appl Phys.

[24] Klimmer A, Ziemann P, Biskupek J, Kaiser U, Flesch M (2009). Phys Rev B.

[25] Osipowicz T, Seng H L, Chan T K, Ho B (2006). Nucl Instrum Methods Phys Res, Sect B.

[26] Sigmund P (1969). Phys Rev.

[27] Ziegler J F, Ziegler M D, Biersack J P (2010). Nucl Instrum Methods Phys Res, Sect B.

[28] Bolse W (1993). Nucl Instrum Methods Phys Res, Sect B.

[29] Prakash J, Tripathi A, Rigato V, Pivin J C, Tripathi J, Chae K H, Gautam S, Kumar P, Asokan K, Avasthi D K (2011). J Phys D: Appl Phys.

